# The kinetic landscape of nucleosome assembly: A coarse-grained molecular dynamics study

**DOI:** 10.1371/journal.pcbi.1009253

**Published:** 2021-07-27

**Authors:** Giovanni B. Brandani, Cheng Tan, Shoji Takada

**Affiliations:** Department of Biophysics, Graduate School of Science, Kyoto University, Kyoto, Japan; Scuola Internazionale Superiore di Studi Avanzati, ITALY

## Abstract

The organization of nucleosomes along the Eukaryotic genome is maintained over time despite disruptive events such as replication. During this complex process, histones and DNA can form a variety of non-canonical nucleosome conformations, but their precise molecular details and roles during nucleosome assembly remain unclear. In this study, employing coarse-grained molecular dynamics simulations and Markov state modeling, we characterized the complete kinetics of nucleosome assembly. On the nucleosome-positioning 601 DNA sequence, we observe a rich transition network among various canonical and non-canonical tetrasome, hexasome, and nucleosome conformations. A low salt environment makes nucleosomes stable, but the kinetic landscape becomes more rugged, so that the system is more likely to be trapped in off-pathway partially assembled intermediates. Finally, we find that the co-operativity between DNA bending and histone association enables positioning sequence motifs to direct the assembly process, with potential implications for the dynamic organization of nucleosomes on real genomic sequences.

## Author summary

Nucleosomes are biomolecular complexes formed by DNA wrapped around histone proteins. They represent the basic units of Eukaryotic chromosomes, compacting the genome so that it fits into the small nucleus, and regulating important biological processes such as gene expression. Nucleosomes are disassembled during disruptive events such as DNA replication, and re-assembled afterwards to preserve the correct organization of chromatin. However, the molecular details of nucleosome assembly are still not well understood. In particular, experiments found that histones and DNA may associate into a variety of non-canonical complexes, but their precise conformation and role during assembly remain unclear. In this study, we addressed these problems by performing extensive molecular dynamics simulations of nucleosomes undergoing assembly and disassembly. The simulations reveal many insights into the kinetics of assembly, the structure of non-canonical nucleosome intermediates, and the influence of salt concentration and DNA sequence on the assembly process.

## Introduction

Nucleosomes are the structural units of chromatin, each consisting of ~147 base pairs of DNA wrapped around a protein histone octamer made of one H3/H4 tetramer and two H2A/H2B dimers [1,2]. Nucleosomes not only enable the efficient compaction of the long Eukaryotic genomic DNA into the nucleus, but also influence key biological process by controlling the access to DNA by other proteins [3], for example during transcription [4]. Such important functions rely on the establishment of precise epigenetic patterns of nucleosome positions, histone variants and histone modifications, which mark the distinction between active and repressed chromatin, and between different genomic regions, e.g., promoters and gene bodies [5]. For example, the electrostatic interactions between histone tails and DNA [6] can be tuned through post-translational modifications such as acetylation [7], controlling nucleosome unwrapping and therefore access to the genome. In order to preserve the identity of each cell, essential in multi-cellular organisms, this genomic organization has to be maintained [8] despite the continuous disassembly of nucleosomes over the course of DNA transcription [9] and replication [10]. However, the molecular details of nucleosome assembly and disassembly have not been yet fully elucidated.

*In vitro* experiments showed that the salt-induced disassembly of nucleosomes starts with DNA unwrapping and it is then followed by the progressive loss of the two H2A/H2B dimers [11], therefore forming firstly an asymmetric hexasome, and finally a tetrasome. Assembly proceeds in the opposite direction, starting with the binding of a H3/H4 tetramer, then followed by the binding of a H2A/H2B dimer on each side [12]. This order of events is due to the specific geometry of the nucleosome (with the H2A/H2B dimers surrounding the central H3/H4 tetramer), and to the more extensive network of protein-DNA hydrogen bonds on H3/H4 relative to H2A/H2B [1,13]. A similar assembly pathway was suggested to occur also *in vivo* [14], except that in this case the process is facilitated by histone chaperones limiting non-nucleosomal histone-DNA interactions [14], or by chromatin remodelers [15]. However, many experiments paint a picture of assembly (or disassembly) more complicated than a simple 2-step process involving tetrasomes, hexasomes and complete nucleosomes, highlighting the existence of various partially-assembled or non-canonical nucleosome structures, such as pre-nucleosomes [16], right-handed nucleosomes [17], remodeler-associated fragile nucleosomes [18], and partially opened nucleosomes [19]. Sub-nucleosomal conformations are also widely distributed across the genome *in vivo* [20], and their formation has been shown to depend in part on the underlying DNA sequence [21]. How such structures form and interconvert between each other remains unclear.

Characterizing the precise molecular details of nucleosome intermediates and the factors controlling their formation, e.g., salt concentration and DNA sequence, would greatly aid our understanding of how chromatin organization is established and maintained. In order to address these questions, we ran extensive coarse-grained molecular dynamics (MD) simulations of nucleosome assembly under different conditions. We consider a simple system made of the strong nucleosome-positioning 601 DNA sequence [22], a H3/H4 tetramer and two H2A/H2B dimers. By analyzing our MD trajectories with Markov state modeling [23], we reveal a complex kinetic landscape characterized by many metastable structures, some of which correspond to previously proposed non-canonical nucleosomes. Our simulations show that A/T nucleosome positioning signals on the 601 DNA sequence direct the binding of the H2A/H2B dimer on the optimal DNA region, so that the dimer is then poised to easily bind the H3/H4 tetramer interface. This effect, which is observed only on the A/T-rich side of the 601 DNA, reveals a highly sequence-dependent assembly process, suggesting a potential mechanism allowing genomic sequences to directly control nucleosome organization *in vivo* [24]. We also investigated the influence of salt on the kinetics, finding that successful nucleosome assembly from a tetrasome is faster at low salt concentration, but that the system is also more likely to remain stuck in off-pathway kinetic traps. This explains the necessity to assemble nucleosomes via salt dialysis [25], or via the addition of chromatin remodelers [16]. The assembly/disassembly mechanism itself is also a function of salt concentration: while at low or intermediate salt the H2A/H2B dimers bind/unbind to/from the H3/H4 tetramer co-operatively with DNA wrapping, unbinding occurs only after DNA unwrapping at high salt. Overall, our results establish clear mechanistic relations between environmental conditions, DNA sequence, and the dynamics of nucleosome assembly.

## Results

### Modeling nucleosome assembly dynamics

In our MD simulations, the DNA is modeled using the 3SPN2.C coarse grained model [26], where each nucleotide is represented by three beads centered on the base, sugar and phosphate groups. The histones are modeled according to the AICG2+ structure-based model [27], where each residue is represented by one bead centered on the Cα atom. This and similar coarse-grained models have been successfully applied to study complex processes such as nucleosome sliding [28–31], unwrapping [7,32,33], and assembly [34], at a fraction of the computational cost required for all-atom simulations [35–39]. Notably, the 3SPN2.C model can capture the sequence-dependent elasticity of DNA [26], and it has been shown to correctly predict the affinity of nucleosome formation for different sequences [33], making this model suitable to investigate the effect of sequence on the assembly kinetics. Further details on the coarse-grained model and its parametrization are provided in the Methods section.

**Fig 1:**
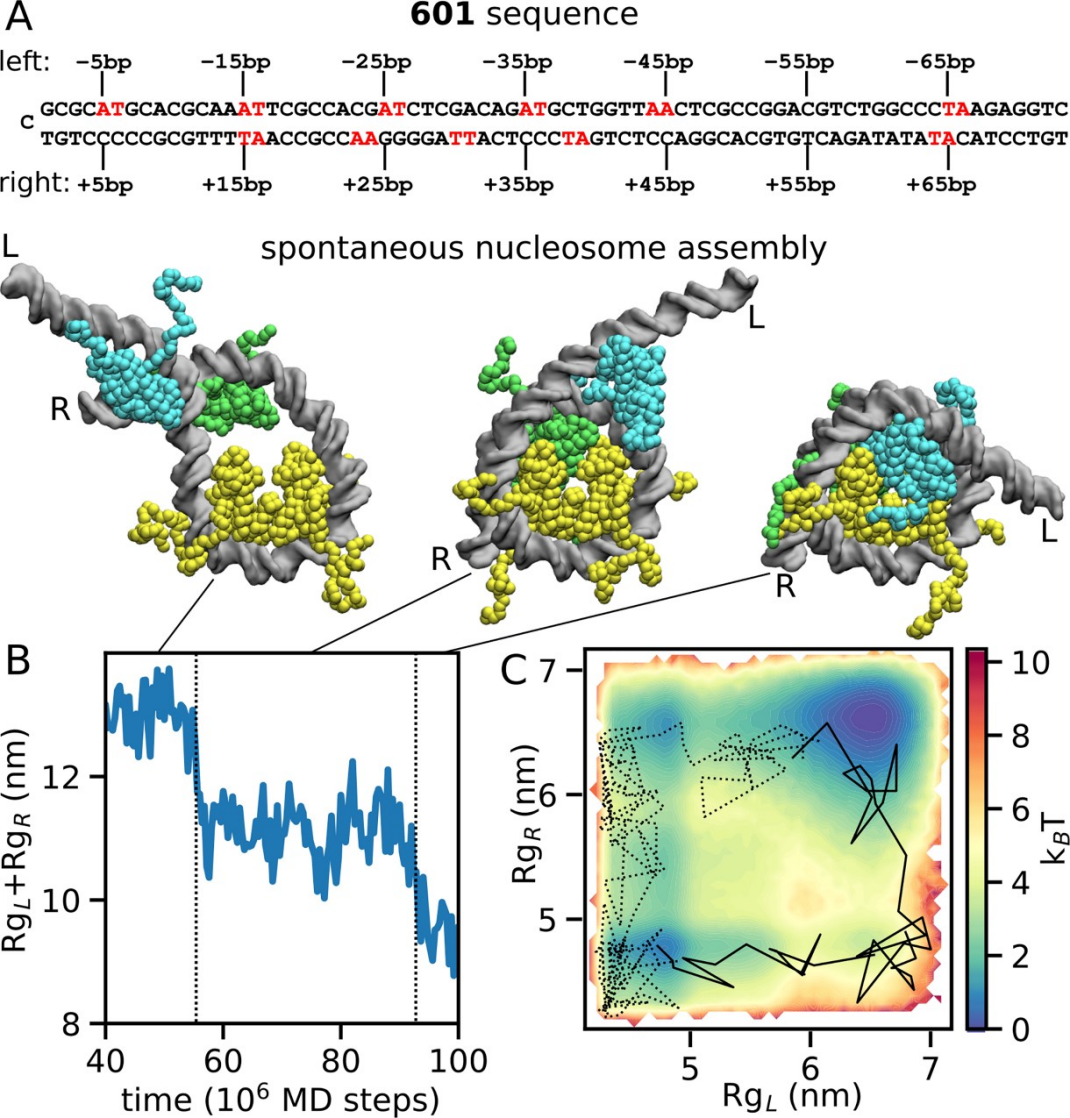
Simulation of nucleosome assembly on the 601-sequence. (A) The 601 DNA sequence. The left (0 to -73 bp) and right (0 to +73 bp) halves are shown on the first and second lines, relative to the dyad position at 0 bp (the left end), highlighting the locations of the A/T periodic motifs in red. (B) Representative nucleosome assembly simulation at 400 mM salt, showing the timeline of the sum of radii of gyration of the left and right halves of DNA (Rg_L_+Rg_R_) during the formation of a complete nucleosome starting from a tetrasome. In the 3 snapshots corresponding to tetrasome (left), hexasome (center) and nucleosome (right), the DNA is shown in grey, the H3/H4 tetramer in yellow, the H2A/H2B dimer binding to the left side of 601 in cyan, and the H2A/H2B dimer binding to the right in green (throughout the paper, the same color code is used). The left and right DNA entry/exits are indicated by the letters L and R respectively. (C) The same trajectory as in panel B (at 400 mM, solid line) projected on the two dimensions Rg_L_ and Rg_R_, together with one successful trajectory at 200 mM (dotted line). The trajectories are superimposed on the free energy landscape at 400 mM calculated by the Markov state model.

Our simulation system consists of the 147-bp nucleosome-positioning 601 DNA [22], one histone H3/H4 tetramer, and two copies of histone H2A/H2B dimers. The 601 sequence was chosen for its widespread use in experiments and for their characteristic positioning motifs asymmetrically distributed around the nucleosome, allowing us to study their role during assembly. Specifically, 10-bp periodic A/T base steps are more frequent on the left side of 601 (called the beta side in Ref. [19], see Fig 1A), leading to enhanced nucleosome unwrapping on the right side relative to the left (i.e. asymmetric unwrapping) [11,40].

We performed nucleosome MD simulations for 10^8^ MD steps at 400 mM mono-valent ion concentration starting from the tetrasome conformation, in which the H3/H4 tetramer is bound on the 601 DNA at the optimal position and both of H2A/H2B dimers are unbound from the tetramer but bound on DNA at different positions depending on the simulation run (see the left snapshot in Fig 1B as an example). Out of 215 runs, we found only one trajectory that reached to the complete nucleosome, which is shown in Fig 1B and S1 Movie. As highlighted by the timeline of the sum of the radii of gyration of the left and right DNA sections (Rg_L_+Rg_R_), in this successful trajectory assembly occurs in two steps: firstly, the right half of DNA bends together with the binding of one H2A/H2B dimer to the tetramer (central cartoon in Fig 1B), then, the left half of DNA folds concomitantly with the assembly of the other H2A/H2B dimer to form the complete nucleosome (right cartoon). In Fig 1C, the same trajectory is projected on the 2-dimensional free energy landscape (obtained by the Markov state model as described below) along the left and right radii of gyration of DNA. The free energy landscape also shows that the left side of the 601 DNA bends more easily (i.e., with a lower free energy cost) than the right side; this asymmetry is discussed later in the results. Other trajectories stopped at various apparently metastable states, due to the limitation of the simulation time.

Starting from the same tetrasome conformations used at 400 mM, we also performed 215 simulations at 200 mM and 300 mM, finding respectively 20 and 7 cases of complete nucleosome assembly (a representative trajectory at 200 mM is shown in Fig 1C). We will discuss the salt concentration dependence in more details later in the results.

### Kinetic landscape of nucleosome assembly via Markov state modeling

In order to characterize the complete landscape and kinetics of nucleosome assembly and disassembly, we generated a Markov state model (MSM) of nucleosome dynamics using 1000 independent simulation runs of 10^8^ MD steps each at 400 mM salt, starting from a diverse set of partially or fully assembled nucleosomes (215 of these are the same described in the previous section, see Methods for more details). The salt concentration of 400 mM was chosen so that neither the fully disassembled nor the fully assembled nucleosome conformations are too much preferred over the others, facilitating the characterization of the dynamics. To build an MSM, we first divide the conformational space into a set of *M* discrete microstates, each corresponding to a group of similar conformations, and then we learn from the unbiased MD trajectories the *M* x *M* transition probabilities to go from each state to another after a certain time interval, the so-called lag-time. Observing either complete assembly or disassembly events in a single trajectory is extremely rare, but the Markov state modeling approach allows us to combine many trajectories together to fully characterize the kinetics of the system, identifying the main long-lived conformations and the dominant pathways of nucleosome assembly. We generated an MSM with *M* = 400 microstates and a lag-time of 4x10^6^ MD steps (see Methods section for more details, and S1 Fig for the convergence of the MSM timescales).

In order to aid the interpretation of the MSM, we can use the PCCA clustering method [41] to further group the 400 microstates into a much smaller number of metastable basins. PCCA provides a hierarchical representation of the model by clustering the conformations into more and more basins by considering progressively faster relaxation processes within the system (associated to the eigenvalues and eigenvectors of the MSM). The simplest, yet meaningful, representation of our system can be obtained using just 4 basins. With this choice, the PCCA method automatically identifies the 4 states that are commonly observed in experiments: the tetrasome, two hexasomes, and the complete nucleosome (representative conformation in S2 Fig). S2 Fig shows that the 4 basins are well separated onto a 2-dimensional projection defined by the two slowest coordinates derived from time-lagged independent component analysis [42] (TICA, see Methods for their definition), further indicating the metastability of these 4 states.

**Fig 2:**
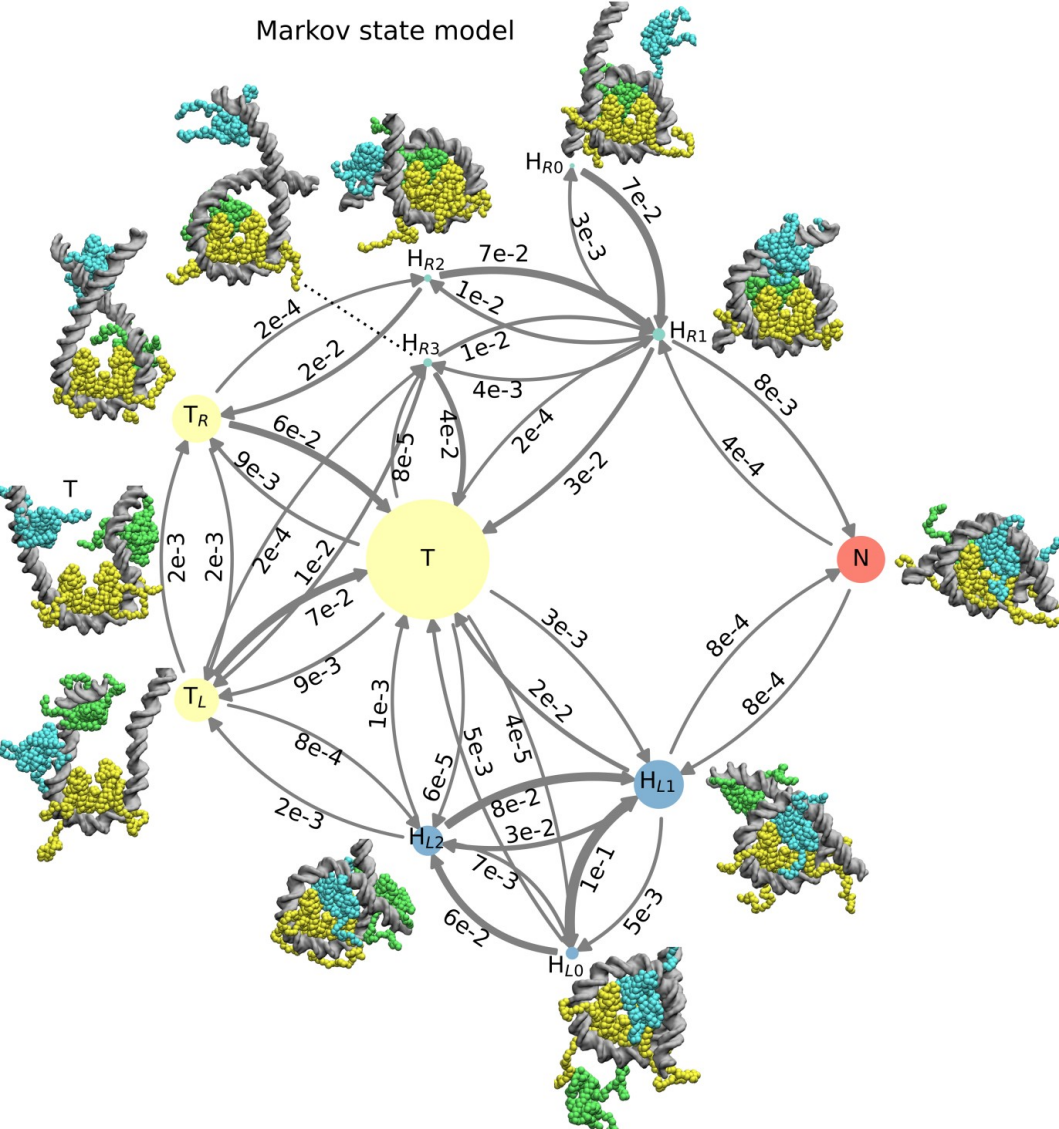
Markov state model of nucleosome assembly. We show the 11 metastable basins identified by PCCA clustering from the MSM at 400 mM, indicating their corresponding representative structures, and the transitions probabilities among the basins. The nodes of the tetrasome (T, T_L_, T_R_), left hexasome (H_L0_, H_L1_, H_L2_), right hexasome (H_R0_, H_R1_, H_R2_, H_R3_), and nucleosome (N) basins, are respectively colored in yellow, blue, cyan and red. The areas of the nodes are proportional to their equilibrium populations. The transition probabilities between basins are represented by the thickness of arrows and the numbers associated (e.g., 3e-2 stands for 3x10^-2^).

However, we can extract more information from our MSM by characterizing a higher number of PCCA basins and the transition probabilities between them. We decided to consider 11 PCCA basins, since this is high enough to provide interesting insights into nucleosome dynamics and low enough to still make the model interpretable. We obtained the transition probabilities between these 11 basins from a coarse-grained hidden Markov state model derived from the full MSM [43]. Fig 2 summarizes the resulting kinetic landscape of nucleosome assembly and disassembly at 400 mM, displaying the identified 11 metastable basins and transition probabilities between these basins (in S3 Fig, we visualize the basins along various combinations of TICA projections). Among the 11 basins, we find one state corresponding to the fully assembled canonical nucleosome (N), seven hexasome states (H_X_) and three tetrasome states (T, T_L_ and T_R_). The three tetrasome states are distinguished by the positions of the unbound H2A/H2B dimers. In the T state one dimer is on the left side of the unwrapped DNA while the other dimer is on the right side, remaining close to the target H3/H4 interface. On the other hand, in the T_L_ and T_R_ states both unbound dimers interact solely with either the left or right side of the unwrapped DNA, respectively. The seven hexasome structures are divided into two types, depending on whether one H2A/H2B dimer is bound to the left (H_L0_, H_L1_ and H_L2_) or the right (H_R0_, H_R1_, H_R2_ and H_R3_) binding interface of the H3/H4 tetramer (respectively wrapping the left or the right sides of the 601 DNA in the canonical nucleosome). Each of these two hexamer types can adopt different conformations depending on the location of the unbound H2A/H2B dimer. In the left hexasome H_L0_, the unbound dimer is either far away or it interacts only weakly with nucleosomal DNA. In H_L1_, the unbound dimer is located on the right side of the DNA relatively close to its optimal location in the complete nucleosome. In H_L2_, the unbound dimer bridges two DNA gyres while facing outside of the nucleosome, in a configuration that cannot easily lead to assembly. H_R0_, H_R1_ and H_R2_ are the respective right hexasome versions of those just described. Finally, in hexasome H_R3_ a H2A/H2B dimer binds to the right interface of the H3/H4 tetramer, but it is stabilized by the wrapping of the left side of the 601 sequence. This metastable configuration is not found on the opposite side (there is no H_L3_ basin), suggesting that it originates from the marked propensity of the left side of 601 to bend. Notably, in the H_R3_ hexasome the DNA follows a right-handed super-helix, contrary to the canonical form that follows a left-handed one. For each of the 11 identified metastable states, we deposited as S1 Data 4 representative coarse-grained nucleosome configurations in PDB format (in S1 Data, which also contains the equilibrium probabilities and the transition probabilities between the basins). Researchers interested in investigating sub-nucleosome conformations in all-atom MD may use these as starting configurations following a previously-developed back mapping procedure [44].

### Asymmetric dynamics of 601 nucleosomes

Our MSM also reveals a clear asymmetry between the left and right side of the 601 sequence. For example, the left hexasome state H_L1_ has an equilibrium probability about 20 times higher than its right counterpart H_R1_, consistent to what observed in past experiments on hexasomes [45]. Kinetics itself is also highly asymmetric: binding always occurs with a higher rate on the left side of 601, whereas unbinding occurs with a higher rate on the right (Fig 2), as observed in past experimental studies [19]. For instance, a tetrasome T transitions into a left hexasome H_L1_ after 4x10^6^ MD steps with probability of ~3x10^-3^, one order of magnitude greater than the probability to observe a T to H_R1_ transition (~2x10^-4^). Similarly, the rate of nucleosome formation is ten times faster when this last assembly step involves H2A/H2B binding on the left side (i.e., from H_R1_ to N).

**Fig 3:**
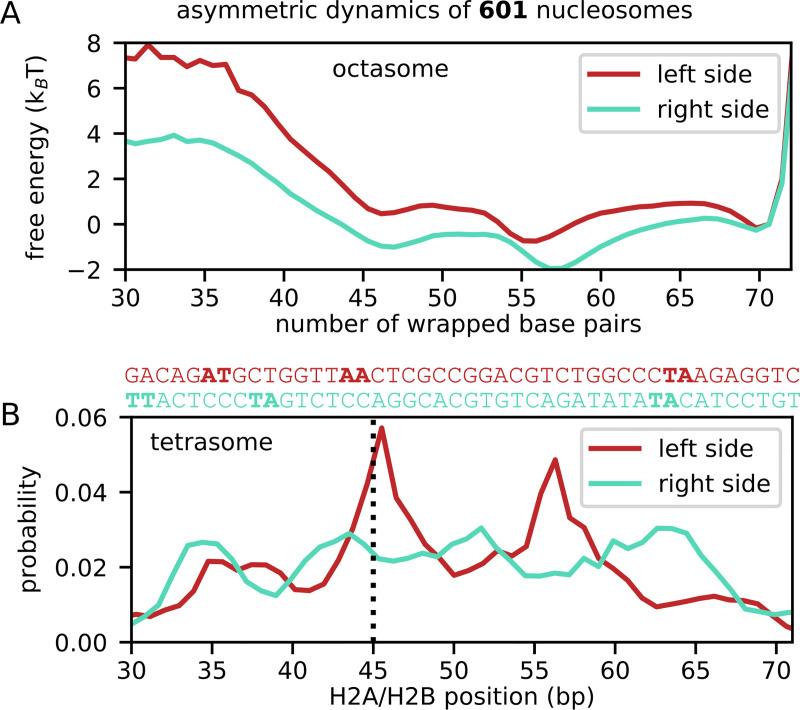
Asymmetric dynamics in 601 nucleosomes. (A) Free energy curves of DNA wrapping for the left (in red) and right (in blue) halves of assembled nucleosome at 400 mM. Only the structural ensemble corresponding to the state N, i.e., complete nucleosome, in the MSM (Fig 2) is used. The free energy at the complete wrapping of 70 bp is set to zero. (B) For the tetrasome states including T, T_R_ and T_L_, we plot the probability of the positions of the unbound H2A/H2B dimers on the left (in red) and the right (in blue) sides of the 601 DNA. The DNA position corresponds to the base pair of the phosphate group that is closest to residue ARG29 on the H2A histone (as described in Methods). On the left side the dimer has a much higher probability to be localized at the same position as when bound to the H3/H4 tetramer (indicated by the black dotted line), facilitating successful assembly. On top of the graph, we align each side of the 601 sequence to the DNA position axis (left side in red, right side in blue).

What is the molecular origin of this kinetic asymmetry? As shown in Fig 1A, the left side of 601 contains periodically spaced A/T base steps at most 5+10n positions from the dyad, whereas on the right side this periodic pattern is much weaker. The importance of the periodic motifs in nucleosome folding stems from the intrinsic bending of the DNA helix, which lowers the energy cost of wrapping around histones [33,40,46]. Indeed, when the histone octamer is assembled (state N), the left side of the 601 DNA has a much higher probability to be fully wrapped than the right side: Fig 3A shows how unwrapping DNA from the left side of 601 (the A/T-rich one) costs ~4 additional k_B_T of energy compared to unwrapping from the right side (the A/T-poor one), similar with what found in another coarse-grained study [46]. Furthermore, even the distributions of H2A/H2B dimer positions along DNA when they are unbound from the tetramer display strong differences (Fig 3B): the left distribution has two sharp peaks separated by 10 base pairs, one of which corresponds to the location found in the left hexasome and complete nucleosome states, while H2A/H2B positioning on the right side of 601 is much more uniform. This shows that the sequence motifs on the left side of 601 (highlighted again in Fig 3B) facilitate the positioning of the H2A/H2B dimer so that it is poised for a successful binding with the H3/H4 tetramer.

### Assembly pathways and salt dependence

The analysis of transition pathways [23] (S4 Fig) in the coarse-grained MSM of the 11 long-lived states shows that two pathways make up most of the successful assembly transitions between the tetrasome T and the full nucleosome N: 66% of the pathways go through the more stable left hexasome H_L1_ (T➔H_L1_➔N), while 19% go through the less stable right hexasome H_R1_ (T➔H_R1_➔N). Therefore, all other hexasome conformations (H_L0_, H_L2_, H_R0_, H_R2_ and H_R3_) do not significantly contribute to successful assembly and may be therefore considered off-pathway kinetic traps.

**Fig 4:**
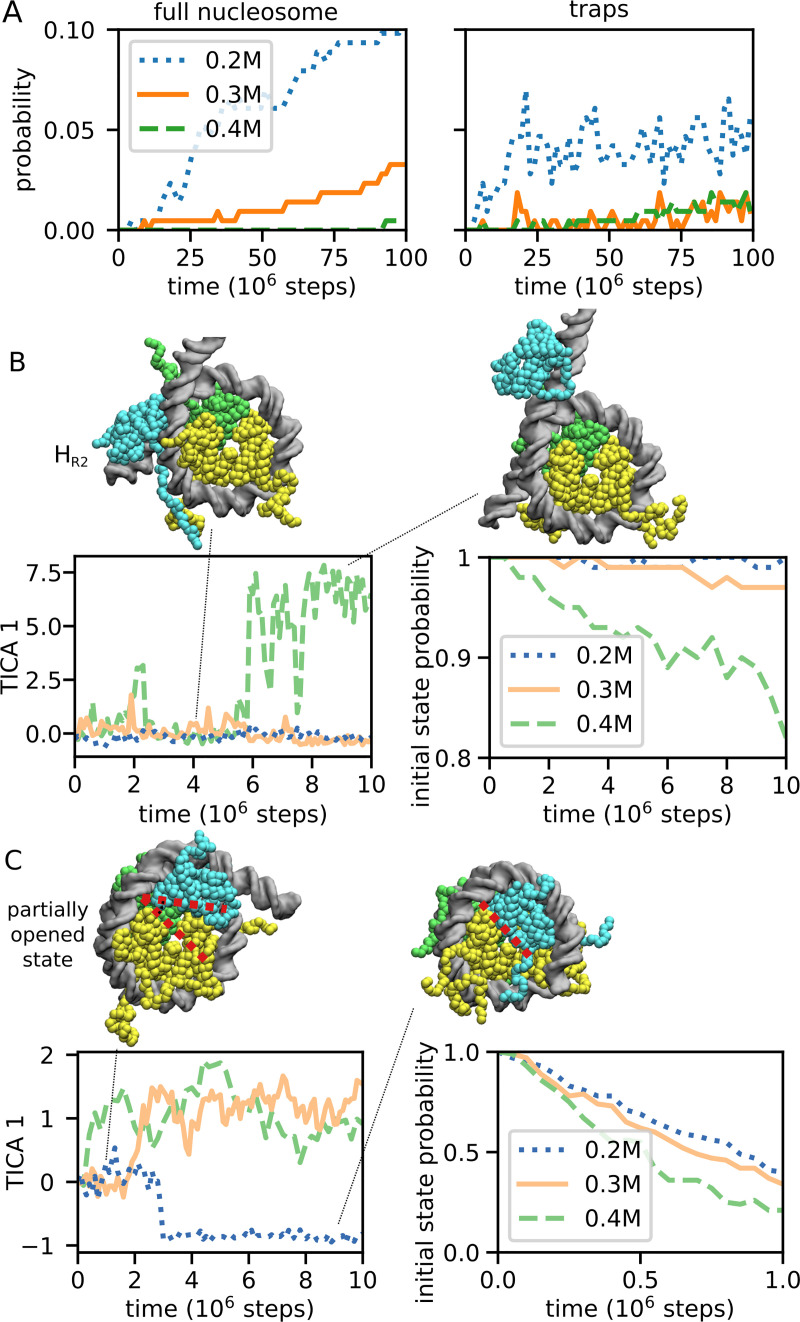
Salt-dependent assembly kinetics and escape from metastable states. (A) The time-dependent probability that, starting from a tetrasome, the system reaches either a full nucleosome (left panel) or one of the kinetic traps (right panel), which are defined as all the off-pathway conformations corresponding to the H_R0_, H_L0_, H_R2_, H_L2_ and H_R3_ basins. (B) The escape kinetics from the H_R2_ kinetic trap. The left panel plots the timeline of the slowest characteristic coordinate, the first coordinate derived from time-lagged independent component analysis [42] (TICA 1, see Methods section for its definition) for a representative trajectory for each salt concentration. The right panel shows, as a function of time, the probability to remain the initial state, defined as the TICA 1 interval from -4 to +4, obtained from 100 trajectories (see Methods for more details). (C) The escape from the partially opened right hexasome identified during the successful hexamer-to-nucleosome assembly events. In order to highlight the conformational change, we indicate with two dotted red lines the wider angle between the unbound H2A/H2B dimer and the neighboring H3/H4 dimer, relative to that found in the fully assembled nucleosome. The left and right panels are the same plot as (B) except that the initial state is defined as the TICA 1 interval from -0.5 to 0.5. The time required to escape from these trapped states increases at low salt.

The above landscape of nucleosome assembly and disassembly was obtained at an intermediate ionic strength (400 mM). However, an interesting question is how the assembly and disassembly processes are affected by changes in salt concentration. As described before, our assembly simulations from the tetrasome configurations at 200 mM, 300 mM, and 400 mM salt resulted in a complete nucleosome assembly in 20, 7 and 1 cases out of 215 runs, suggesting that low salt favors assembly. This is reasonable, since nucleosome are stabilized by protein-DNA electrostatic interactions. However, it does not explain the observation that the optimal procedure to assemble nucleosomes *in vitro* is by slowly decreasing the salt concentration from high to low values [25], and otherwise chromatin remodelers are required to successfully complete assembly [16]. One possibility is that at low ionic strength the kinetic landscape becomes more rugged, and the system struggles to escape from off-pathway traps. Indeed, Fig 4A indicates that although lower salt concentration increases the probability to reach the complete nucleosome, it also significantly increases the probability to reach the kinetic traps identified from the MSM (H_L0_, H_L2_, H_R0_, H_R2_ and H_R3_).

To further study this effect, we run for each ionic strength 100 10^7^-steps simulations starting from a H_R2_ hexasome conformation, one of the traps that do not participate in the assembly, where the unbound H2A/H2B dimer bridges both left and right sides of DNA. Consistently with our hypothesis, the time required to escape from the initial state increases as we lower the salt concentration (Fig 4B). This analysis was performed by plotting the MD trajectories along the slowest TICA coordinate (see Methods for more details).

During the successful assembly trajectories at 200 mM and 300 mM salt, we also found evidence of new metastable states that are not clearly captured by our MSM at 400 mM. In these conformations the nucleosome is already very compact but partially opened: due to the absence of DNA bending at super-helical location +/-3 (i.e., 3 DNA helical turns away from the dyad), one or both H2A/H2B dimers are unbound from the tetramer and oriented at a wider angle relative to the canonical conformation (see snapshot in Fig 4C). During our trajectories, we found this angle to vary between ~10 and ~50 degrees (the angle was computed based on the orientation of the longest helices in histones H2A and H4). PDB coordinates of representative partially opened conformations (part of the H_L_, H_R_ or T states) have been deposited in S1 Data together with the other metastable states defined from the MSM. To further investigate their properties, we run 100 10^7^-steps simulations starting from the partially opened right hexasome at 200, 300, and 400 mM salt. Fig 4C shows that, similarly to the H_R2_ state, the escape from the partially opened state becomes slower as we decrease the salt concentration, presumably because this conformation is stabilized by electrostatic interactions of the H2A C-terminal and H2B N-terminal histone tails bridging the two DNA gyres. Furthermore, at 300 mM we find that starting from a partially opened hexasome conformation the probability to transition either into the complete nucleosome or into a more open hexasome is about 50%, similarly to a transition state (within 5x10^6^ MD steps, we observe complete assembly events in 269 runs out of 400 starting from the left partially opened state, and in 156 out of 400 from the right one). Finally, we note that all the evidence points to these partially opened nucleosomes being the same as those identified in a recent FRET study [19]. In both experiments and in our simulations, 1. the angle between the open H2A/H2B dimer and the tetramer very similar (a value of ~20 degrees was suggested from experiments), 2. the stability of these states decreases with increasing ionic strength, and 3. they are intermediates along the nucleosome assembly pathways. In order to test the accuracy of our predictions, it is also useful to compare the transition rates between complete nucleosomes and opened states, which are also available from FRET experiments [19]. In principle, one MD timestep in our simulations equals 15 fs; however, coarse-graining smoothens the free energy landscape, speeding up the dynamics, so we map 1 MD timestep to 30 ps, based on the speedup observed in our previous work on nucleosome sliding using the same model [29] (a similar speedup was also observed in coarse-grained simulations of protein folding [47]). Experimentally, at low salt concentration, the characteristic times for the transitions (inverse rates) from the complete nucleosome to the partially opened state and back have been estimated to be respectively >50 ms and >3 ms, with an equilibrium constant of 0.06. From the simulations at 300 mM, we estimated the inverse rates to be respectively ~5x10^8^ MD steps = 15 s and ~10^6^ MD steps = 30 ms (see Methods), consistent with the lower bounds from experiments, and an equilibrium constant of ~0.002, somewhat underestimating the stability of the partially opened state.

**Fig 5:**
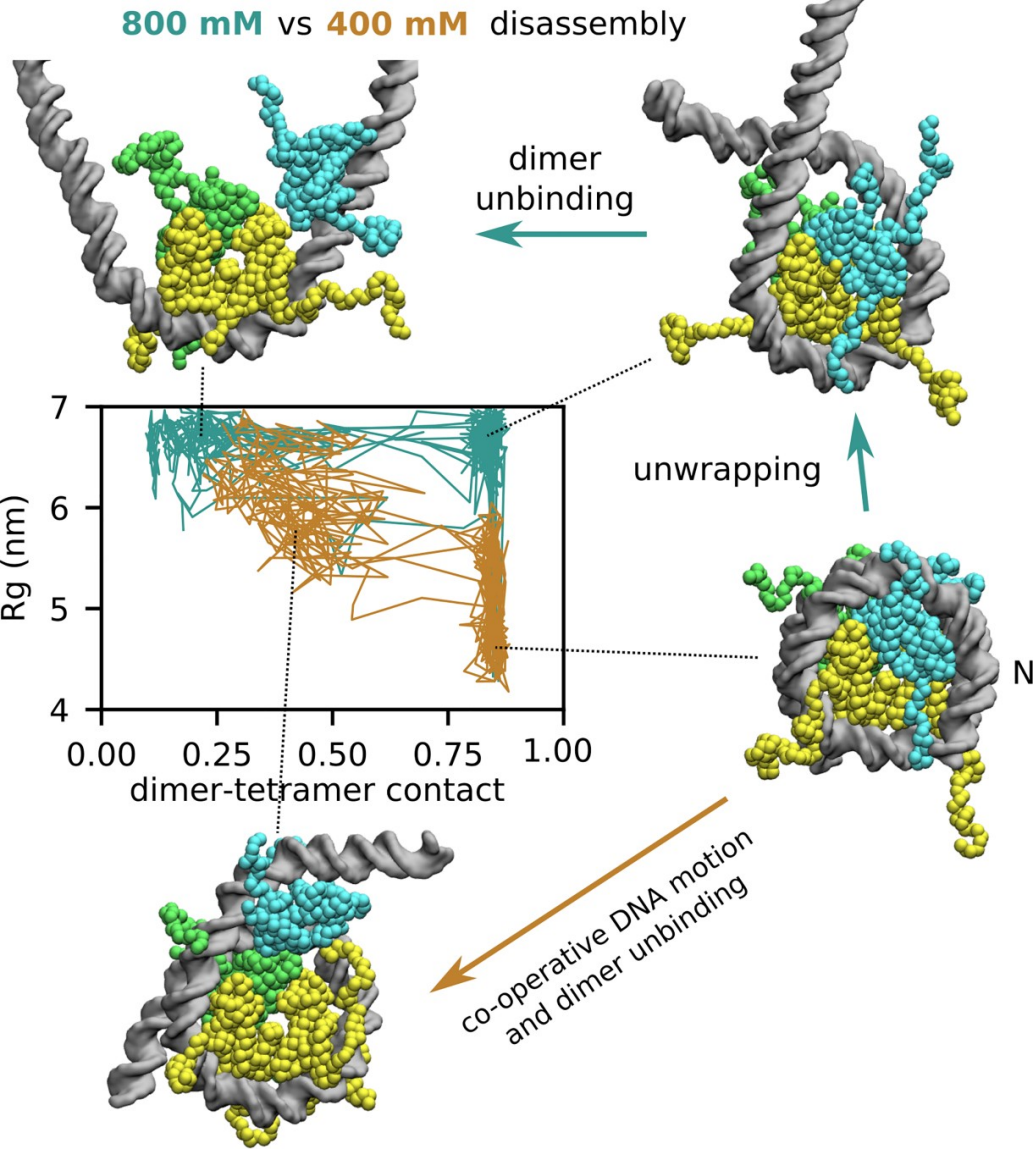
Salt-dependent nucleosome disassembly pathways. We project a set of representative disassembly trajectories along the DNA radius of gyration (Rg, the vertical axis) and a coordinate capturing dimer-tetramer contact formation (the horizontal axis, see Methods for the precise definition), at either 400 mM (in brown) or 800 mM salt (in cyan). While at intermediate salt concentration disassembly occurs in a single step involving dimer unbinding from the tetramer cooperatively with the stretching motion of DNA, at high salt concentration dimer unbinding follows a significant unwrapping of DNA from the histone octamer.

In order to explore whether the disassembly mechanism is also affected by the salt concentration, we run additional 10^8^-step simulations starting from the complete nucleosome at 500, 600, 700, and 800 mM salt (30 trajectories each). As expected, nucleosomes become unstable at high salt, with frequent DNA unwrapping and dimer unbinding. Interestingly, disassembly pathways appear qualitatively different at intermediate (400 mM) and high (800 mM) salt: while in the former case H2A/H2B unbinding from the tetramer proceeds cooperatively with the unwrapping of DNA, in the latter case DNA unwrapping anticipates histone unbinding (Fig 5). This trend is in qualitative agreement with the experimental literature: the cooperative transition is consistent with the recent results obtained by FRET at intermediate salt [19], while the non-cooperative behavior was suggested by a combination of SAXS and FRET at very high 1.9 M salt concentration [11].

## Discussion

In conclusion, our coarse-grained MD simulations revealed the complex landscape of nucleosome assembly and its modulation by salt and DNA sequence. Firstly, we used Markov state modeling to summarize the assembly dynamics on 601 sequences at 400 mM salt concentration (at which the assembly is reversible). Our MSM highlighted, apart from the expected canonical nucleosome, tetrasome and hexasome states, several long-lived configurations characterized by alternative interactions between histones and DNA. Interestingly, in one of the right hexasome states (H_R3_), a canonical histone hexamer wraps the DNA in a right-handed way. Right-handed nucleosomes can form in experiments after the application of positive DNA supercoiling [48,49] and are also found *in vivo* within centromeres [17]. However, while past works investigated how such change in DNA handedness could originate from an equivalent change in the arrangement of the histone folds [48–50], our simulations predict that these right-handed structures can also form via an alternative wrapping of the DNA around a canonical histone hexamer, without requiring a complex rearrangement of histones [48]. Since this state is found only on one side of 601, the simulations further suggest that right-handed nucleosomes are stabilized by specific DNA sequences. Torsional stresses, such as those generated by the passage of RNA polymerase, are also expected to affect the stability of various nucleosome configurations, as found in experiments on nucleosomes with super-coiled DNA [51], and in a recent MD study that investigated the effect of torsion on nucleosomal DNA unwrapping [52].

Our simulations also provide fresh insights into asymmetric nucleosome dynamics on 601 DNA. This sequence is characterized by several nucleosome positioning motifs, namely A/T base steps periodically spaced every 10 bp, which favor the wrapping of DNA around histones. However, these motifs are asymmetrically distributed along the sequence, favoring H2A/H2B dimer assembly on the left side of 601 by about one order of magnitude. Asymmetric disassembly of nucleosomes along 601 was observed in many experiments [11,19,40]; our MD simulations revealed that DNA sequence motifs promote assembly by directing the binding of H2A/H2B dimers towards the H3/H4 target, an effect originating from the co-operativity between histone binding and DNA bending at low salt concentration. We suggest that by promoting the assembly of nucleosomes directly at highly flexible genomic regions, while avoiding stiff DNA regions such as poly-A tracts [53], the sequence dependence in the rates of assembly may also favor the maintenance of nucleosome positioning *in vivo* after disruptive events such as replication. The importance of the sequence-dependent shape and flexibility of DNA for protein-DNA recognition [54] and chromatin organization [55] has been well investigated in the literature, but our simulations further reveal previously unappreciated dynamical effects. In the future, we plan to investigate the dynamics of nucleosomes on real genomic sequences to further explore these ideas. For example, yeast promoters could be an ideal target, since yeast nucleosome positions have been mapped with base-pair accuracy [56], and the relationship between nucleosome organization and DNA flexibility along the genome has been recently investigated experimentally [24]. Studying a large number of sequences at the same time should also allow us to derive general features of the sequence-dependent nucleosome dynamics that are not evident from the analysis of only one or few sequences.

Finally, our simulations highlighted how salt dependence can have a large effect on the kinetics of the system. The MSM generated at 400 mM salt shows that successful nucleosome assembly pathways are relatively simple: the dominant pathway from the tetrasome (T) to the complete nucleosome conformation (N) goes through a single intermediate hexasome state with a H2A/H2B dimer bound to the H3/H4 tetramer on the left side of 601 (H_L1_), while the second dominant pathway goes through the opposite intermediate hexasome (H_R1_) where the dimer is bound to the tetramer on the right side of 601. All other metastable states do not significantly participate into the assembly process, effectively acting as kinetic traps slowing down nucleosome formation. While low salt favors assembly, we find that it also stabilizes off-pathway kinetic traps, explaining the importance of salt dialysis for the successful assembly process [25]. Under some conditions, experiments found that histones and DNA form non-canonical pre-nucleosome structures that require chromatin remodelers to be converted into the canonical forms [16]. We note that the experimental observation that pre-nucleosomes may form on just ~80 bp of DNA [16] mirrors our simulation results showing that metastable non-canonical nucleosomes generally display less interactions with DNA than those found in the canonical form. Based on our findings, we predict that pre-nucleosomes should resemble the haxasome states H_L2_ and H_R2_, where the unbound H2A/H2B dimer bridges the two opposite ends of nucleosomal DNA to prevent successful assembly unless some large rearrangement first occurs.

Simulations at 200 and 300 mM also revealed critical intermediate states along the successful pathways of nucleosome assembly: here the nucleosome is almost completely formed, but one H2A/H2B dimer does not yet make direct contacts with the H3/H4 tetramer, with the two interfaces at a wider angle relative to the canonical nucleosome. These partially opened nucleosomes where also recently identified in a series of FRET experiments [19]. Simulations indicate that these partially opened states are roughly halfway along the nucleosome pathways at low salt (i.e., they are transition states); in virtue of this, modulating the stability of this state, for instance by histone chaperones or epigenetic modifications, should have a large effect on assembly and disassembly rates, suggesting its potential role *in vivo*. Again, we found that the formation of these structures does not require any conformational change within histones (which may nevertheless play a role in nucleosome dynamics [57]), but are instead stabilized by non-specific electrostatic interactions between histone tails and DNA. In addition to conformational stability, salt concentration also affects the assembly/disassembly pathway itself: while at low and intermediate salt the H2A/H2B dimer binds the H3/H4 tetramer co-operatively with DNA bending (so that DNA sequence can directly affect kinetics), at high salt these two processes are uncoupled, with DNA unwrapping anticipating dimer-tetramer dissociation. These results recapitulate past experimental observations under various conditions [11,19].

Overall, using MD simulations in combination with the MSM analysis, we made a series of important predictions on nucleosome dynamics: some of them already verified in experiments (the intermediate partially opened nucleosome state, the asymmetric dynamics on 601, and the salt-concentration-dependent assembly pathways), and others waiting to be tested in the future (the detailed conformations of off-pathway nucleosome states such as pre-nucleosomes and right-handed nucleosomes, and the role of sequence-dependent nucleosome kinetics for chromatin organization). The position coordinates of the metastable nucleosome conformations deposited in S1 Data will also represent a useful resource for researchers interested in further investigating nucleosome assembly, for instance by aiding the design of FRET experiments [19], or by providing starting configurations for all-atom MD simulations through a back-mapping procedure [44]. Finally, we envision that our coarse-grained model will be of great use to characterize nucleosome assembly in more realistic scenarios typical of *in vivo* experiments, for example in the presence of RNA polymerase [58], histone chaperones [50], and remodelers [15].

## Methods

### Coarse-grained model

To enable the complete study of nucleosome assembly and disassembly, we utilize coarse-grained molecular modeling: we map each amino-acid to a single bead [27], and each nucleotide to three beads corresponding to base, sugar, and phosphate groups [26]. The functional potentials and force-field parameters are chosen according to the AICG2+ structure-based model for histones [27] and the 3SPN2.C sequence-dependent model for DNA [26]. The flexible histone tails (residue ids 1–32 for H3, 1–23 for H4, 1–14 and 121–128 for H2A, 1–26 for H2B) are modeled according to a sequence-dependent statistical local potential specifically designed for disordered regions, which was derived by Boltzmann inversion from the distribution of angles and dihedral angles observed in the non-structured subset of the Protein Data Bank [59]⁠. The potential of the remaining folded regions is based on the native structures of the first copies of each H3/H4 and H2A/H2B dimers present in the nuclesome crystal structure with PDB id 1KX5 (using a single reference for each pair of dimers ensures the symmetry of the histone octamer, which is important to study the asymmetry induced by DNA sequence). Whenever two protein residues *i* and *j* are within a certain cutoff distance in the native structure, the coarse-grained potential includes a contact interaction that favors the binding of the two residues [27]:

$$V_{ij}^{\mathrm{native}}=\epsilon_{ij}\left[5{\left(\frac{r_{0}^{ij}}{r^{ij}}\right)}^{12}-6{\left(\frac{r_{0}^{ij}}{r^{ij}}\right)}^{10}\right]$$

Where ε_ij_ is a contact-dependent energy determined from the all-atom reference structure and r_0_^ij^ is the native distance between the residues. In addition, the protein potential also includes bond, angle and dihedral interactions with equilibrium values set according to the native structure, and non-native excluded volume interactions proportional to the 12^th^ power of the distance.

Experimentally, in the absence of DNA or at high salt the histone octamer breaks into a H3/H4 tetramer and two H2A/H2B dimers [11,19,60], but using the default AICG2+ potential we do not observe histone octamer breakage under these conditions. This happens because the default AICG2+ scaling factor defining the overall strength of the residue-residue interactions was optimized for studying the folding of single-domain proteins, but in our case the attraction between the H3/H4 and H2A/H2B dimers is over-estimated. In order to find a suitable scaling factor for the native interactions between H3/H4 and H2A/H2B residues, we scanned different values performing MD simulation at either low salt (200 mM), at which we expect our nucleosomes to be stable, or high salt (800 mM), at which we expect disassembly. We find that scaling factors lower than 0.2 lead to unstable nucleosomes even at low salt, whereas for values higher than 0.4 we do not observe disassembly at even at high salt. Rescaling the dimer-tetramer interactions by a factor of 0.3 reproduces the expected behavior of nucleosomes: out 30 independent simulation runs of 10^8^ MD steps starting from a fully assembled nucleosome, we observed no disassembly events at 200 mM salt, whereas we observed disassembly in 4 out of 30 runs at 800 mM; as shown by the MSM reported in the Results section, nucleosome assembly is reversible at intermediate 400 mM salt, with comparable equilibrium populations of tetrasomes and complete nucleosomes. This critical concentration at which nucleosomes disassemble is somewhat lower than the value observed in experiments, which is around 600 mM [19]; however, taking into account the high computational cost of the parameterization, we regard the agreement between experiments and simulations satisfactory, and sufficient to provide reliable insights into the nucleosome assembly dynamics. We use the dimer-tetramer scaling factor of 0.3 in all simulations reported in this study.

Histones and DNA interact via excluded volume, Debye–Hückel electrostatics, and hydrogen bond interactions. The hydrogen bonds are modeled according to a distance- and angle-dependent potential based on nucleosome crystal structures, and was calibrated based on nucleosome structural stability and DNA unwrapping [28]. The size of the beads to define excluded volume interactions, and the parameter settings for the hydrogen bonds are the same used in our previous works [28–30]. The charges on the histone residues are determined via the RESPAC method [61], via which we optimized the coarse-grained Debye–Hückel electrostatic potential against the one obtained by solving the full Poisson-Boltzmann equation around the all-atom histone core structures, similarly to what done in other coarse-grained models of the nucleosome [62] (the optimization was performed at 100 mM salt, but the resulting charges are very robust this choice). The RESPAC procedure was applied to the globular part of the H3/H4 tetramer and the two H2A/H2B dimers, whereas for the flexible tails we used the standard integer charges on the Lysine, Arginine, Aspartic and Glutamic acids residues only.

### Simulation setup

All reported molecular dynamics (MD) simulations have been performed with the software CafeMol [63] by integrating the equations of motion using Langevin dynamics at 300 K with a timestep of 0.3 CafeMol time units (corresponding to 15 fs). During the MD simulations, we apply harmonic restraints on the structured region of the two H3 histones (0.001 kcal/mol/Å^2^) and on the phosphate beads of the DNA dyad residues (bp id 73, 0.1 kcal/mol/Å^2^), limiting large-scale sliding of DNA relative to the H3/H4 tetramer (but not the unbound H2A/H2B dimers). The nucleosome sliding mechanism was already analyzed in previous studies [28,29]⁠, and especially for the 601 positioning sequence considered here can be extremely slow. In our simulations, the H3/H4 is always placed at its optimal experimental position on the DNA sequence; preventing its sliding allows to avoid exploring less favorable positions and to focus solely on the assembly dynamics, which is our main focus. Finally, the system is enclosed into a spherical volume with a radius of 20 nm by a harmonic potential. CafeMol input files to run nucleosome assembly simulations are provided in the S2 Data.

The starting conformations for the 1000 MD simulation runs at 400 mM used to generate the MSM have been obtained during high-salt (800 mM) simulations starting from complete nucleosomes, and then selecting 1000 timeframes that include tetrasomes (215 conformations), left and right hexasomes (105 and 118 conformations, respectively), and nucleosomes (562 conformations). To quantify the salt-dependence of the nucleosome kinetics, we also performed, at both 200 and 300 mM salt, 215 10^8^-steps simulation runs starting from the 215 tetrasome conformations. To analyze the escape from 2 metastable states (either the H_R2_ hexasome or the partially opened state) as a function of salt concentration, we run 100 independent 10^7^-steps trajectories starting from the same conformation (either H_R2_ or partially opened H_R1_) at 200, 300, and 400 mM salt. In order to characterize the time necessary to escape from these states (analysis in Fig 4), we projected the molecular positions onto the slowest TICA motions [42] within each basin. The TICA projection was performed separately for the H_R2_ and partially opened metastable basins based on the positions of H2A residues 46 to 73 of the unbound dimer and the left side of DNA (the one interacting with the unbound H2A/H2B dimer) during the simulations at 300 mM salt. The inverse transition rate from the partially opened state to the complete nucleosome at 300 mM was estimated from the 100 simulations starting from the partially opened state by fitting an exponential curve to the survival probability to remain in the initial state before transitioning into the complete nucleosome, while the inverse transition rate for the opposite process was obtained from 100 10^7^-MD-steps simulations starting from the complete nucleosome, during which we observed two transitions into the partially opened state.

### Markov state modeling and analysis of metastable states

To generate the Markov state model of nucleosome assembly at 400 mM salt from our 1000 MD simulations, we first have to cluster the configurations into discrete states based on a distance between configurations defined on a suitable space. To this aim, we project the particle positions onto a series of collective variables that capture all the relevant modes of nucleosome dynamics: H2A/H2B dimer binding to DNA and to H3/H4, DNA wrapping, and DNA handedness. Since the two H2A/H2B dimers are identical, we generated variables that are symmetric under an exchange of these two dimers. In principle, the two H3/H4 binding interfaces are also identical, but the 601 DNA that is wrapped around the tetramer is not symmetric, so we do want to distinguish between the left and right H3/H4 interfaces, corresponding respectively to the dimer binding the left or the right side of the 601 DNA. We defined 5 types of collective variables (for a total of 4+4+2+2+1 = 13 collective variables):

- For each H3/H4 tetramer interface, the sum and the maximum value of the contacts with the two H2A/H2B dimers. A dimer-tetramer contact is defined as c = 1/(1+d/σ), where σ = 1 nm and d is the distance between the center of mass of H2B residues 73–100 and the center of mass of H4 residues 65–92 (corresponding to the two interfacial regions). Therefore, a contact takes a value close to 1 when a H2A/H2B dimer binds the H3/H4 tetramer in its native configuration, whereas it is close to 0 when the H2A/H2B dimer is far from the H3/H4 tetramer. We first compute the 4 contacts between each of the 2 tetramer interfaces (t1 and t2) with the two dimers (d1 and d2): c_t1,d1_, c_t1,d2_, c_t2,d1_, and c_t2,d2_. Then, we define the 4 collective variables: c_t1,d1_+c_t1,d2_; c_t2,d1_+c_t2,d2_; max(c_t1,d1_,c_t1,d2_); and max(c_t2,d1_,c_t2,d2_).
- For each side of the DNA (left, residue ids 1–73, and right, ids 75–147), the sum and the maximum value of the contacts with the two H2A/H2B dimers. A DNA-dimer contact is defined as 1/(1+d/σ), where σ = 1nm, and d is the distance between residue ARG29 on H2A (in the canonical nucleosome this residue binds the DNA at SHL +/-4.5) and the closest DNA phosphate group. The sum of the DNA-dimer contacts enables us to distinguish between the various tetrasome conformations where two dimers can bind either the same (states T_L_ and T_R_ in Fig 2 of the main text) or two different DNA regions (state T). The maximum value of the contact distinguishes strong or weak binding of the H2A/H2B dimer to the DNA.
- For each side of the DNA (left and right), the H2A/H2B dimer location along the DNA, which is defined as the residue id of the DNA phosphate group (on the 5’ strand for either side) that is closest to the H2A ARG29 residue on the dimer closest to the considered DNA side. For example, for the location on the left side of the DNA, if the first H2A/H2B dimer is closer than the second one (based on the minimum distance above), we consider the base pair id of the phosphate group closest to the first dimer. This variable enables us to capture the sliding of the H2A/H2B dimers along the DNA when they are unbound from the H3/H4 tetramer.
- For each side of the DNA, the number of base pairs wrapped around the histones, where a base pair is considered unwrapped if the center of mass of its base groups is displaced radially from the nucleosome super-helix axis by more than 6 Å.
- The handedness of the nucleosome, which is captured by the component of the distance vector between the centers of mass of base pairs with ids -35 and +35 (relative to the dyad id 0) along the nucleosome super-helix axis. Positive values correspond to canonical left-handed nucleosomes, whereas negative ones correspond to right-handed conformations such as those in the metastable state H_R3_ in Fig 2 of the main text.

Starting from these 13 collective variables, we aggregate all 1000 MD trajectories and perform a further dimensionality reduction using time-lagged independent component analysis (TICA) [42] as implemented in PyEMMA 2 [64]⁠ using a lag-time of 10^6^ MD steps. We then select the top 8 slowest coordinates (which explain 95% of the kinetic variance in the data) and cluster the conformations into 400 discrete states using k-means [65]⁠. The MSM was then generated by PyEMMA 2 [64] using a lag-time of 4x10^6^ MD steps. Further increasing the lag-time does not lead to significant changes in the implied time scales of the MSM, indicating the robustness of the results (S1 Fig). The TICA co-ordinates are also ideally suited for visualization purposes: in S2 Fig we show the first two coordinates are sufficient to separate the system into 4 regions corresponding to nucleosomes, left and right hexasomes and tetrasomes (more TICA coordinates can further separate into more metastable basins with faster transitions among them, S3 Fig).

## Supporting information

S1 Fig MSM convergence. Implied time scales of the MSM as a function of the chosen lag-time. For the final model, we use a lag-time of 4x10^6^ MD steps, after which most of the implied time scales have essentially converged. The shaded regions indicate 95 confidence intervals.
(PDF)

S2 Fig 4 PCCA basins. The nucleosome conformations belonging to the 4 PCCA metastable basins projected along the first two slowest TICA coordinates used to generate the MSM (each color corresponds to one basin). Both PCCA and TICA clearly separate the system into 4 distinct regions corresponding to complete nucleosomes, left and right hexasomes, and tetrasomes. The first TICA is proportional to the number of contacts between the H3/H4 tetramer and the H2A/H2B dimers, whereas the second TICA is proportional to the difference between the right and left dimer-tetramer contacts. 4 representative conformations are shown next to the corresponding basin region.
(PDF)

S3 Fig 11 PCCA basins. Same as S2 Fig but using 11 PCCA metastable basins. In this case, more TICA coordinates are necessary to further separate the nucleosome conformations into the basins.
(PDF)

S4 Fig Transition pathways. We show the metastable basins and the two top pathways to reach the complete nucleosome N starting from the tetrasome T. Both pathways involve a single intermediate state corresponding to one of the two caninical hexasomes (H_L1_ or H_R1_). Above the arrows we indicate the percentage of pathways passing through this transition. The x coordinate corresponds to the committor probability as computed from the MSM, which is defined as the probability to reach state N before coming back to state T.
(PDF)

S1 Movie Nucleosome assembly. Representative assembly trajectory at 400 mM starting from a tetrasome conformation, the same shown in Fig 1B in the main text.
(MP4)

S1 Data Coordinates of metastable states. For each of the 11 metastable states identified from our MSM at 400 mM using PCCA clustering, we deposited 4 representative coarse-grained nucleosome conformations in PDB format. The conformations are named “state*conf#.pdb”, where * corresponds to the state name as described in the text (T, T_L_, T_R_, H_L0_, H_L1_, H_L2_, H_R0_, H_R1_, H_R2_ and H_R3_, N), and # goes from 1 to 4. The file “msm_hmm11.dat” contains the equilibrium probabilities of the various basins and the transition probabilities between them. The data also contain 4 representative partially opened conformations for each of the left hexasome, right hexasome, and tetrasome states, named “partially-opened_*_conf#.pdb”, where * is H_L_, H_R_ or T and # goes from 1 to 4.
(ZIP)

S2 Data MD simulation setup. Example CafeMol input files used to run nucleosome assembly simulations as described in the Methods section.
(ZIP)
