# Supplementary figures and images for: The kinetic landscape of nucleosome assembly: A coarse-grained molecular dynamics study
Source: PLoS Comput Biol. 2021 Jul 27;17(7):e1009253. doi: 10.1371/journal.pcbi.1009253 (PMC8345847; doi:10.1371/journal.pcbi.1009253)

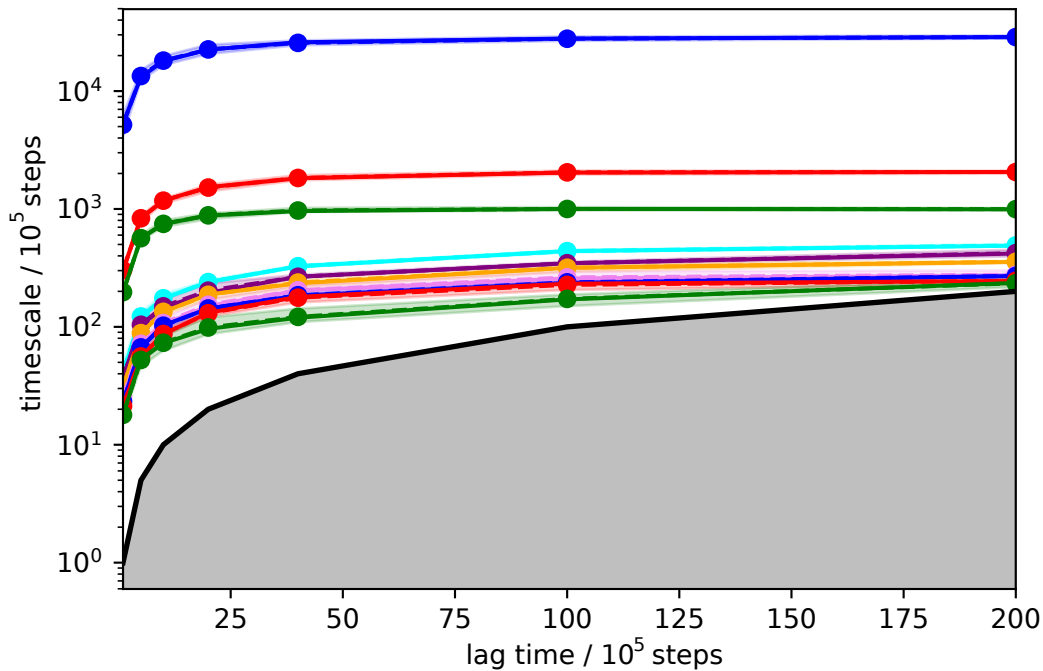

Supplement: S1 Fig — Implied time scales of the MSM as a function of the chosen lag-time. For the final model, we use a lag-time of 4x10^6^ MD steps, after which most of the implied time scales have essentially converged. The shaded regions indicate 95 confidence intervals. (PDF) [file pcbi.1009253.s001.pdf]

TICA 2

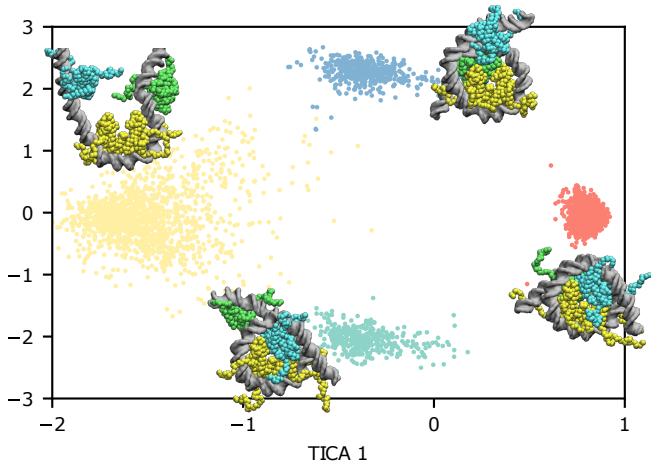

Supplement: S2 Fig — The nucleosome conformations belonging to the 4 PCCA metastable basins projected along the first two slowest TICA coordinates used to generate the MSM (each color corresponds to one basin). Both PCCA and TICA clearly separate the system into 4 distinct regions corresponding to complete nucleosomes, left and right hexasomes, and tetrasomes. The first TICA is proportional to the number of contacts between the H3/H4 tetramer and the H2A/H2B dimers, whereas the second TICA is proportional to the difference between the right and left dimer-tetramer contacts. 4 representative conformations are shown next to the corresponding basin region. (PDF) [file pcbi.1009253.s002.pdf]

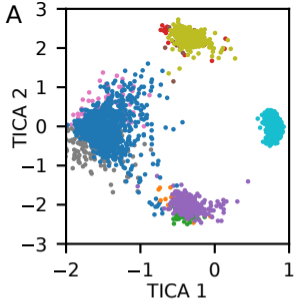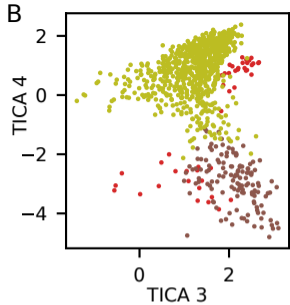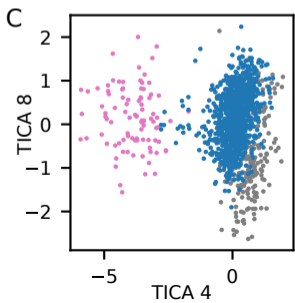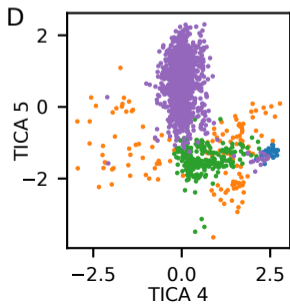

Supplement: S3 Fig — Same as S2 Fig but using 11 PCCA metastable basins. In this case, more TICA coordinates are necessary to further separate the nucleosome conformations into the basins. (PDF) [file pcbi.1009253.s003.pdf]

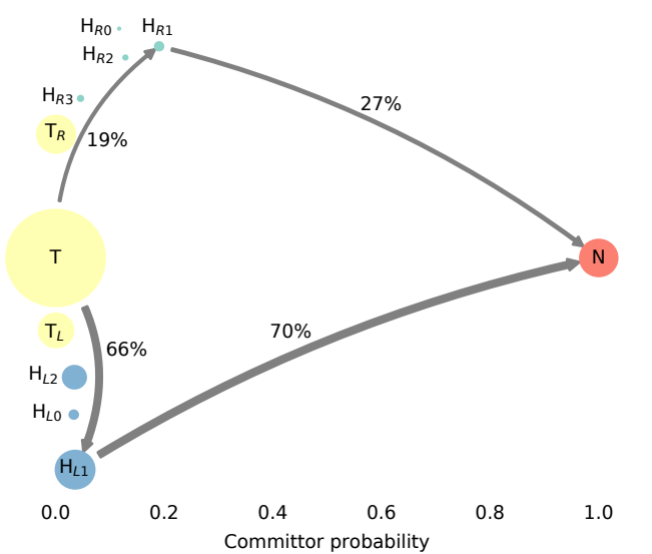

Supplement: S4 Fig — We show the metastable basins and the two top pathways to reach the complete nucleosome N starting from the tetrasome T. Both pathways involve a single intermediate state corresponding to one of the two caninical hexasomes (HL1 or HR1). Above the arrows we indicate the percentage of pathways passing through this transition. The x coordinate corresponds to the committor probability as computed from the MSM, which is defined as the probability to reach state N before coming back to state T. (PDF) [file pcbi.1009253.s004.pdf]
